# Genesis of interictal spikes in the CA1: a computational investigation

**DOI:** 10.3389/fncir.2014.00002

**Published:** 2014-01-27

**Authors:** Shivakeshavan Ratnadurai-Giridharan, Roxana A. Stefanescu, Pramod P. Khargonekar, Paul R. Carney, Sachin S. Talathi

**Affiliations:** ^1^J Crayton Pruitt Family Department of Biomedical Engineering, University of FloridaGainesville, FL, USA; ^2^Department of Otolaryngology, Kresge Hearing Research Institute, University of MichiganAnn Arbor, MI, USA; ^3^Electrical and Computer Engineering, University of FloridaGainesville, FL, USA; ^4^Department of Pediatrics, University of FloridaGainesville, FL, USA; ^5^Qualcomm Corp R&DSan Diego, CA, USA

**Keywords:** interictal spikes, hippocampal CA1 region, computational models, paroxysmal depolarization shift, temporal lobe epilepsy

## Abstract

Interictal spikes (IISs) are spontaneous high amplitude, short time duration <400 ms events often observed in electroencephalographs (EEG) of epileptic patients. *In vitro* analysis of resected mesial temporal lobe tissue from patients with refractory temporal lobe epilepsy has revealed the presence of IIS in the CA1 subfield. In this paper, we develop a biophysically relevant network model of the CA1 subfield and investigate how changes in the network properties influence the susceptibility of CA1 to exhibit an IIS. We present a novel template based approach to identify conditions under which synchronization of paroxysmal depolarization shift (PDS) events evoked in CA1 pyramidal (Py) cells can trigger an IIS. The results from this analysis are used to identify the synaptic parameters of a minimal network model that is capable of generating PDS in response to afferent synaptic input. The minimal network model parameters are then incorporated into a detailed network model of the CA1 subfield in order to address the following questions: (1) How does the formation of an IIS in the CA1 depend on the degree of sprouting (recurrent connections) between the CA1 Py cells and the fraction of CA3 Shaffer collateral (SC) connections onto the CA1 Py cells? and (2) Is synchronous afferent input from the SC essential for the CA1 to exhibit IIS? Our results suggest that the CA1 subfield with low recurrent connectivity (absence of sprouting), mimicking the topology of a normal brain, has a very low probability of producing an IIS except when a large fraction of CA1 neurons (>80%) receives a barrage of quasi-synchronous afferent input (input occurring within a temporal window of ≤24 ms) via the SC. However, as we increase the recurrent connectivity of the CA1 (*P*_sprout_ > 40); mimicking sprouting in a pathological CA1 network, the CA1 can exhibit IIS even in the absence of a barrage of quasi-synchronous afferents from the SC (input occurring within temporal window >80 ms) and a low fraction of CA1 Py cells (≈30%) receiving SC input. Furthermore, we find that in the presence of Poisson distributed random input via SC, the CA1 network is able to generate spontaneous periodic IISs (≈3 Hz) for high degrees of recurrent Py connectivity (*P*_sprout_ > 70). We investigate the conditions necessary for this phenomenon and find that spontaneous IISs closely depend on the degree of the network's intrinsic excitability.

## 1. Introduction

Mesial temporal lobe epilepsy (MTLE) is a chronic neurological disease that affects the hippocampus and the inner regions of the temporal lobe. MTLE is characterized by recurrent seizures (ictal activity) and interictal spikes (IISs), which typically occur in between seizure epochs in the form of transient discharge events which are clearly discernible from background EEG activity. Studies involving long-term EEG monitoring in animal models of MTLE show that IISs also occur prior to the first instance of spontaneous ictal activity (Buzsáki et al., 1991). In chronic *in vivo* animal models of MTLE, it has been observed that IISs start within a few weeks after initial brain injury and steadily increase in frequency of occurrence (Buzsáki et al., 1991). Despite an overwhelming evidence for an IIS as a characteristic observable feature in EEG of MTLE patients (Engel, 1996), the role of IISs and its clinical manifestation in MTLE remain unclear. For example, while there is evidence to suggest that IISs interfere with normal cognition and learning (Holmes and Lenck-Santini, 2006; Kleen et al., 2010) and may facilitate the development of spontaneous seizure activity (Staley et al., 2005), recent *in vitro* experiments suggest that an increase in interictal spiking activity may serve as an anti-epileptogenic agent (Avoli et al., 2006). In order to completely understand the role of IISs in MTLE, we need to study the effects of selectively invoking or suppressing IISs on demand. Progress in this direction will most certainly first require a fundamental understanding of the network mechanisms underlying the generation of an IIS in an epileptic brain.

In MTLE, IISs are thought to originate from the CA3/2 region of the hippocampus involving a group of pacemaker pyramidal (Py) cells (Jefferys, 1990; Wittner and Miles, 2007). IISs propagate as population bursts throughout the CA3 subfield and on to the CA1 subfield via the Schaffer collaterals (SC) (Stoop and Pralong, 2000). A number of *in vivo* and *in vitro* studies have demonstrated that when the SC fibers are cut or the CA3 removed, CA1 loses its ability to generate IISs (Lewis et al., 1990; Stoop and Pralong, 2000). While the CA3 may be necessary for the initiation of IISs in the hippocampus, the CA1 subfield is critical for propagating the IIS to subcortical brain structures outside the hippocampus via the subiculum and the entorhinal cortex (Lopes da Silva et al., 1990; van Groen and Wyss, 1990; Dvorak-Carbone and Schuman, 1999). Furthermore, in MTLE, the CA1 is one of the first hippocampal subfields that undergoes rapid morphological and structural changes, such as recurrent pyramidal axonal sprouting and neuronal cell death (Lehmann et al., 2000). It is therefore essential to understand how the morphological and structural changes implicated in the CA1 subfield of an MTLE brain influence the subfields ability to exhibit IISs in response to afferent input from the SC.

**Figure 1:**
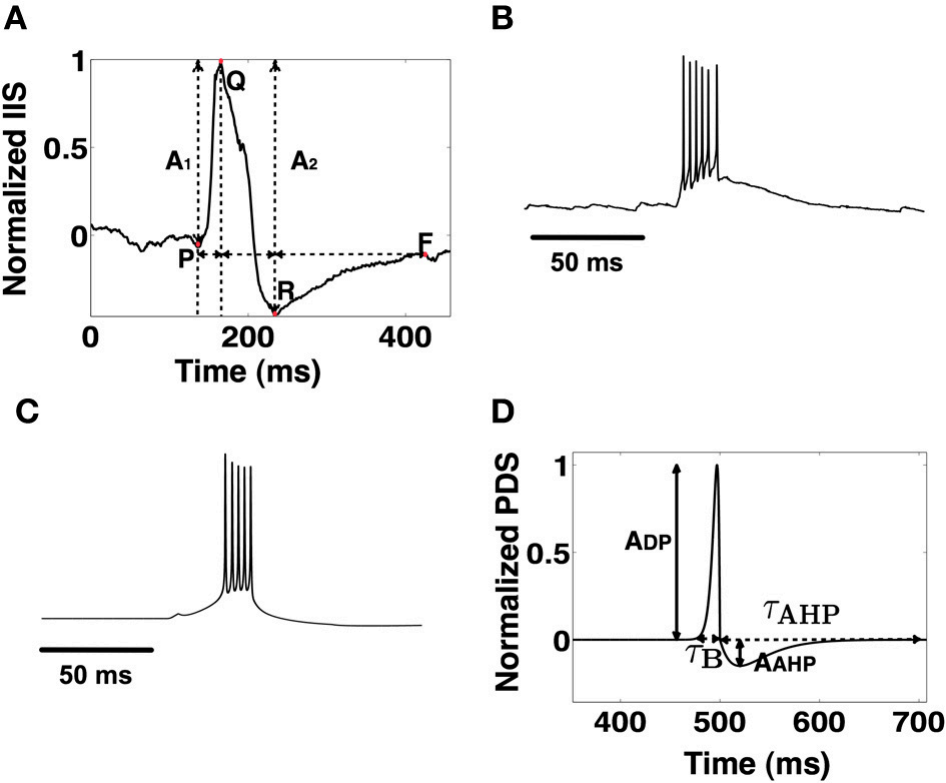
**(A)** A typical IIS observed in experimental EEG recordings. The measurable amplitude features (*A*_1_ and *A*_2_) and temporal features (Δ_*PQ*_,Δ_*QR*_, and Δ_*RF*_) are used to validate candidate IIS. **(B)** An experimentally recorded PDS event from rat CA1 tissue *in vitro*. **(C)** Model generated PDS using the Golomb CA1 pyramidal cell model. **(D)** A template PDS that defines the envelope of a burst defined by parameters τ_*B*_ (burst width) and τ_AHP_ (AHP width). The template model of PDS is used to determine PDS and synchronization parameters for the formation of an IIS.

The cellular correlate for an IIS is the epileptiform bursting activity of Py cells commonly referred to as the paroxysmal depolarization shift (PDS) (McCormick and Contreras, 2001; Staley and Dudek, 2006). The PDS represents a large (20–40 mV), long lasting (50–200 ms) neuronal depolarization which results in the initiation of high frequency burst of action potentials (200–300 Hz) (Kandel et al., 2000). The depolarization wave is usually followed by a slow afterhyperpolarization (AHP). An example of PDS recorded from resected hippocampal tissue of a TLE patient is shown in Figure 1B. The PDS phenomenon is attributed to a number of factors including increased extracellular *K*^+^ concentration, reduced extracellular *Ca*^2+^ concentration (Yaari et al., 1983;, Formenti et al., 2001; Burgo et al., 2003; Smith et al., 2004; Golomb et al., 2006), increased synaptic drive (Jefferys, 1990) and channelopathies (McNamara, 1994). In the pathological CA1 Py cell population, the duration of a PDS burst its AHP can have variable durations. Furthermore, the PDSs themselves can occur with varying degree of synchronization (Netoff and Schiff, 2002). Identifying the correspondence between the features of these cellular events and the extent of their synchronization is critical for exploring their role in the formation of IISs in the CA1.

The primary goal of this study is to develop a biophysically relevant computational network model of the CA1 subfield in order to investigate the network mechanisms implicated in the formation of IISs within the subfield. Using experimental data on IISs recorded from an *in vivo* animal model of chronic limbic epilepsy, we first ask the following question: what are the characteristics of PDS events that are implicated in the generation of an experimentally observable IIS? We develop a method for analyzing recorded IISs in order to empirically estimate the underlying PDS characteristics. These include the depolarization time interval, the hyperpolarization duration of a typical PDS event and the degree of synchronization between these PDS events. This data is used to tune a synaptically reduced neuronal network model in order to enable the model to generate PDSs with features matching those obtained using the empirical estimation procedure. The tuning procedure allows us to estimate the relative strengths of the excitatory and inhibitory neuronal populations implicated in the generation of PDS events in the minimal network model. This information in addition to data from literature (Kandel et al., 2000; Demont-Guignard et al., 2009) is used to build a bio-physically relevant network model for the CA1 subfield. We then use this model to investigate the following specific questions: (1) How does the formation of an IIS in the CA1 depend on the degree of sprouting (recurrent connections) between the CA1 Py cells and the fraction of CA3 Shaffer collateral (SC) connections onto the CA1 Py cells? and (2) Is synchronous afferent input from the SC essential for the CA1 to exhibit IIS? Our results suggest that the CA1 network is capable of eliciting IIS activity primarily through two mechanisms of network synchronization: (1) input-induced synchronization, where the CA1 network with low intrinsic excitability characterized by low degree of recurrent connections between the CA1 Py cells can elicit IIS in response to synchronized barrage of afferent input from the SC with high degree of SC to Py connectivity and (2) emergent synchronization, where the CA1 network with high degree of recurrent connections between the excitatory Py cells can elicit spontaneous IIS activity even in response to asynchronous afferent input from SC.

A major motivation for identifying the various conditions under which the CA1 can exhibit IIS, is to eventually develop a control strategy to disrupt IIS events. Any control strategy should take into consideration the fact that different network conditions may require different control schemes to disrupt IIS. This affects not only the control approach but also possibly the choice of actuation used in the implementation of a control system. We anticipate that the CA1 network model presented here and our findings of the general conditions under which the CA1 can elicit an IIS response could serve as a computational tool to effectively investigate and develop various control paradigms for the ultimate purpose of controlling IIS.

## 2. Materials and methods

### 2.1. Experimental setup

Adult male Sprague Dawley rats (*n* = 3) of age 63 days and weighing between 200 and 265 g were used for the experiments. Thirty-two microwire recording electrodes were bilaterally implanted into the CA1 region of each rat's hippocampus. Chronic limbic epilepsy was induced in the rats using the *in vivo* self-sustaining electrical status epilepticus animal model (Lothman et al., 1989). The Institutional Animal Care and Use Committee of the University of Florida approved all protocols and procedures (IACUC protocol D710). The rats were housed in a controlled environment and monitored with continuous video and CA1 local field potential recordings. At the end of the recording session, the rats were sacrificed and the intact brains were excised. The isolated intact brains were imaged with high strength magnetic resonance microscopy to confirm the location of the electrode placement within the CA1 region of the hippocampus (Talathi et al., 2009). Data from a single electrode implanted in the contral-lateral CA1 subfield was analyzed to extract IIS shape profiles (Talathi et al., 2009), which were detected using a modified spike clustering algorithm (Fee et al., 1996) that sorted spikes into separate clusters.

Data for reference PDSs were obtained from Dr. K. Srinivasa Babu (Christian Medical College, Vellore, See Acknowledgments). Ventral hippocampal horizontal slices of 400 μM thickness were dissected out from a 4 week old Wistar rat. The slices were transferred into an interface chamber containing artificial CSF with 118 mM NaCl, 2.5 mM KCl, 2 mM CaCl_2_, 2 mM MgCl_2_, 25 mM NaHCO_3_, 1.24 mM NaH_2_PO_4_, and 10 mM glucose equilibrated with dissolved Carbogen (95% O2 and 5% Co_2_) at room temperature and at a pH of 7.4. After 1 h of incubation, the slices were perfused with 20 μM Bicuculline to induce epileptic bursting. Recordings were performed with Glass electrodes (4–8 mOhm) fabricated from borosilicate glass capillaries, filled with pipette solution containing (in mM) 135 Kmeso_4_, 8 NaCl, 10 HEPES, 2 Mg_2_ATP, and 0.3 Na_3_GTP. Signals were digitized at 10 KHz and recorded using Clampex software (Molecular devices, USA).

### 2.2. Computational setup

All computational networks were built using a custom C++ framework. The code was compiled using g^++^ (ver 4.4.6) and run using a RHEL 6 cluster. Up to 64 simulations could be run in parallel using the OpenMPI framework. The computational cluster consisted of 2 Intel Xeon dual-quad core dual-rackmounts. This provided 32 cpu cores. The total RAM available to the system was 48 GB. Networks of 300 neurons for a simulation time of 1 s, typically took between 10 and 30 min depending on the number of synapses implemented for a given network. Analysis of experimental data, simulation data, and template based studies were done on an Intel Xeon quad core PC using Matlab. Numerical integration was performed using the fourth order Runge-Kutta(RK4) algorithm with a time step of 0.01 ms. The RK4 technique was used with delay-differential equations (See Methods: Synaptic model) after verifying that there were no noticeable deviations in simulation results with the Euler method of numerical integration. We compare the CA1 model's output for different integration methods and different time steps in the Supplemental data section. The codes used for producing some of the results will be made available on modelDB.

### 2.3. Neuron models

In this section we introduce the neuronal models that we employ for the construction of our network. We use single compartment, standard Hodgkin-Huxley type neuronal models for both the pyramidal cells (Py) and interneurons. For interneurons, we model the predominant populations found in the CA1 which are the basket (B) and orien/alveus (OA) cells. The Py cells are modeled using the Golomb neuron model (Golomb et al., 2006), the B cells are modeled using the Wang-Buzsaki model (Wang and Buzsáki, 1996), and the OA cells are modeled using the Wang neuron model (Wang, 2002). The neuron models are mathematically structured as follows:

(1) $$\begin{array}{l} c\dot{V}=I_{DC}-I_{g}(V,n)-I_{KCa}(V,Ca^{2+})+I_{\text{syn}}\\ \dot{n}=G(V,n)\\ m_{\infty }=U(V) \end{array}$$

where, *c* is the membrane capacitance, *V* is the membrane voltage, *I*_*g*_ represents the sum of the currents flowing due to voltage gated intrinsic membrane ion channels (Na, K, Ca etc.), *I*_*KCa*_ is the membrane current due to calcium gated potassium channels, *I*_syn_ is the total synaptic current and *I*_*DC*_ is an intrinsic current that sets the cell's excitability. **n** ∈ [0, 1] and **m**_∞_ ∈ [0, 1] are the gating variables vectors for ion channels present on the neuron membrane with finite rise time constants and instantaneous rise-time kinetics, respectively. We provide the details on the neuron models channel currents and kinetics in the Supplemental data section.

### 2.4. Synaptic model

The synaptic current contribution for all the neuron models is modeled as: *I*_syn_(*t*) = *g*_*s*_ *S*(*t*)(*V* − *E*_*s*_), where *g*_*s*_ can be the α-Amino-3-hydroxy-5-methyl-4-isoxazolepropionic acid (AMPA) or γ-Aminobutyric acid (GABA) synaptic conductance, *E*_*s*_ is the reversal potential of the synapse (approximately 0 mV (Andrasfalvy and Magee, 2001) for AMPA and −72 mV for GABA Cohen et al., 2002), and *S*(*t*) represents the fraction of bound synaptic receptors and has the following form:

(2) $$\dot{S}(t)=\frac{S_{0}(V_{pre}(t-\tau_{x}))-S(t)}{\hat{\tau }(S_{1}-S_{0}(V_{pre}(t-\tau_{x})))}$$

where

(3) $$S_{0}(V_{pre})=0.5(1+\tanh(120(V_{pre}-0.1)))$$

where *V*_*pre*_(*t*) is the pre-synaptic neuronal spike, τ_*x*_ is the synaptic delay for synapse type *x* ∈ {Py,B,OA}. τ_*Py*_ was assumed to be near instantaneous at 0.5 ms. Basket cells were assumed to have a larger delay of 5 ms due to their distance from Py cells. OA cells that were the furthest away from Py cells had synaptic delays of 10 ms with Py cells. The delay between OA and B was 5 ms. The delay from synapses of interneurons was calculated assuming a conduction velocity of 0.1 m/s (Salin and Prince, 1996) and considering the average distance between neuronal populations. The rise and decay time constants of the synaptic current is expressed as $\tau_{R}=\hat{\tau }(S-1)$ and $\tau_{D}=\hat{\tau }S$, respectively. τ_*R*_ for all cells was assumed to be near instantaneous at 0.1 ms. τ_*D*_ for Py, fast firing B and slower OA synapses were set at 1 ms, 3 ms, and 5 ms, respectively (Geiger et al., 1995; Bartos et al., 2002, 2007; Taxidis et al., 2012).

In Table 1 we list the values of the synaptic strength conductances used for the CA1 model. This was done by matching the post-synaptic potentials (PSPs) of various neurons with physiological recordings (Cobb et al., 1997; Ali et al., 1998; Taxidis et al., 2012). As we were unable to obtain physiological data for PSPs of the synaptic connections for Py-Py and SC-B, we assumed SC-B strengths to be the same as other inhibitory synapses and the Py-Py excitatory strength was assumed to be the same as SC-Py's. We estimated the strength of SC-Py using empirical techniques (see Results: Estimating the synaptic parameters of the CA1 network).

**Table 1:** **Values of *g*_*s*_ used based on matching of PSP amplitudes with physiological data**.

	**g_*s*_ (nS)**	**Model PSP (mv)**	**Reported PSP (mv)**
PY-B	0.1	1.24	1.4
PY-OA	0.1	1.33	1
B-PY	0.5	0.65	0.45
OA-PY	0.5	0.65	0.46
B-B	0.5	0.71	0.25

*The resting membrane potential of the neurons in the CA1 network model from which the PSPs were measured were: −65.73 mV for Py cells, −64.02 for B cells, and −61.54 mV for OA cells*.

### 2.5. Detailed CA1 network model

**Figure 2:**
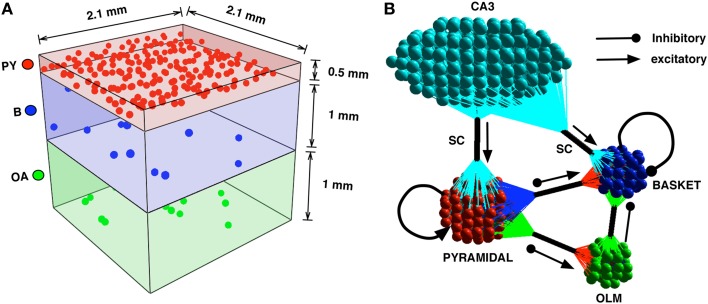
**(A)** The CA1 neurons in the network model are distributed in cuboid layers. The Py cells are distributed in a thinner layer representing the stratum pyramidale. The interneurons B and OA are distributed in slightly thicker layers, with the basket cells being in closer proximity to the pyramidal cells than the OA. **(B)** The connectivity is described by the clusters of colored spheres representing neuron groups and their synapse bundles (black cylinders). The arrows with triangular heads represent incoming excitatory connections and those with circular heads represent incoming inhibitory projections.

We construct a detailed representation of a section of the CA1 using information interpreted from literature (Kandel et al., 2000; Andersen et al., 2006; Demont-Guignard et al., 2009). Following from Demont-Guignard et al. (2009), we construct the CA1 network with 80%−20% excitatory-inhibitory neuron ratio using 225 Py cells, ~22 B cells, and ~22 OA cells that are distributed in a 3D cuboid of 0.21 × 0.21 × 0.21 mm^3^ as shown in Figure 2A. Each of the neuron types are distributed uniformly within their individual cuboid layers. The connection between any given pair of cells depends on the euclidean distance between the pairs and follows a Gaussian distribution with standard deviation σ_*x*,*y*_, where x and y are the pair of connected cells. We initially choose σ_*Py*,*Py*_ = 20 μm such that the probability of the connection between any two Py cells is low. The other parameters for the network are taken from Demont-Guignard et al. (2009) as: σ_*Py*,*B*_ = 166.6 μm, σ_*B*,*Py*_ = 233.3 μm, σ_*Py*,*OA*_ = 166.6 μm, σ_*OA*,*Py*_ = 280 μm, σ_*B*,*B*_ = 233.3 μm, σ_*OA*,*B*_ = 280 μm. OA cells do not exhibit recurrent inhibition and B cells do not synapse on to the OA cells. The CA3's input to the CA1 via SC is simulated as a barrage of action potentials through AMPA synapses that synapse on to Py and B cells. 100% of the B cells each receive an SC synapse, while the percentage of Py cells each receiving SC input can be varied (Typically 70%). We illustrate the connectivity of the CA1 network in Figure 2B.

Since IISs are a hyper-excitable phenomenon, it is necessary to ensure that the CA1 network is in a hyper-excitable state. In the Results section, we have provided details on estimating synaptic conductances that are conducive for this purpose. We also increase the Py cell's excitability by setting the *I*_*DC*_ value of Pyramidal cells to 0.3 nA/cm^2^. We additionally set *I*_*DC*_ = −0.3 nA/cm^2^ for OA cells to prevent them from firing spontaneously and inhibiting the Py cells. Bio-physically this can be interpreted as due to decreased interneuronal cell activity. The effects of sprouting in the network model was a critical component in this paper. We define the degree of sprouting (*P*_sprout_) by the average number of incoming synapses from other Py cells that each Py cell receives. For example, *P*_sprout_ = 40 corresponds to an average of 40 input synapses from other Py cells to every Py cell. We define *P*_sprout_ = 100 to be the upper-limit in our simulations due to the observations in our results that IIS frequency saturates at this value of sprouting (see Results: IIS formation as an induced or emergent synchronization phenomenon).

### 2.6. Local field potential measurement

The local field potential (LFP) refers to extracellular voltage signals (≤500 Hz) recorded sub-durally from the brain(Dzhala and Staley, 2003). EEG level signals are usually found in the range of ≤100 Hz while signals from multiunit recordings are found at around ≥300 Hz. Both these components are usually present in various combinations in LFP depending on the type of electrode and site of the electrode placement (Dzhala and Staley, 2003). For our computational model we focus on the lower frequency EEG-range signals as IISs fall in the EEG-range signal category. To model this, we must be able to capture transmembrane potentials that might contribute to an IIS (Buzsáki et al., 2012). Researchers seeking to reproduce LFP signals in a computational environment commonly consider summed synaptic current contributions. This however may not be sufficient to capture detailed aspects of highly synchronized events such as IIS. Transmembrane potentials can build up significantly under synchronous conditions to affect extracellular measurements (Buzsáki et al., 2012). These include fast spikes(in bursts) and AHP. When there is an inflow of cations from the extracellular medium into the neuron, a current source is created extracellularly. To maintain electro-neutrality, there will be an outflow of ions into the extracellular field creating a sink. A dipole created in such a manner obeys Kirchoff's current law. An LFP's main component is from the dipoles (Buzsáki et al., 2012) whose measured potential varies with the inverse square of distance between the current source-sink site and the recording electrode position. Recent studies have shown that there may also be significant contributions from monopoles whose creation does not follow Kirchoff's law (Destexhe and Bedard, 2012; Riera et al., 2012). However, the effects of monopoles on recorded LFP from the brain are not completely understood. Hence, we choose not to model our LFP based on monopoles. With these factors in mind, we choose to model the LFP as a direct function of the membrane voltage of individual Py neurons. Similar approaches have also been used by other researchers such as Ursino and La Cara (2006).

(4) $$LFP(t)=\sum_{i}^{N}\frac{V_{si}(t)}{r_{i}^{2}}$$

*N* is the total number of pyramidal cells, *V*_*si*_ is the *i*th neuron's soma membrane voltage, and *r*_*i*_ is the distance of the *i*th neuron from the measuring electrode. In our network model, the LFP electrode is placed in the vicinity of the stratum pyramidale layer. This arrangement indicates that the major LFP component will be from the pyramidal soma.

## 3. Results

### 3.1. Template based analysis of the temporal profile of PDS events

We begin by identifying the temporal characteristics of PDS events and the degree of PDS synchronization that is implicated in the generation of an experimentally observed IIS. In Figure 1A, we show an example of a typical IIS (normalized with peak value of 1) recorded from the CA1 subfield of an epileptic rat (Talathi et al., 2009). In agreement with data from earlier works (Jayakar et al., 1989; Adjouadi et al., 2005), the IIS profile exhibits the following empirical characteristics: (a) The total duration Δ*t* of the IIS, Δ*t* = Δ_*RP*_ + Δ_*PF*_ + Δ_*FQ*_, is between 50 ms and 400 ms. (b) The two half waves (R to P and P to F) satisfy the condition that their absolute difference is less than or equal to their calculated average (Jayakar et al., 1989): |Δ_*RP*_ − Δ_*PF*_| ≤ 0.5(Δ_*RP*_ + Δ_*PF*_) (c) The downward deflection voltage *A*_2_, is larger than the upward deflecting voltage *A*_1_, satisfying the condition: 0.25 ≤ *R* ≤ 2, where *R* = *A*_1_/*A*_2_.

An example of a typical PDS, obtained from a rat hippocampal slice perfused with Bicuculline (see Methods) is shown in Figure 1B. In general, there is a large variability in the shape profile of PDSs (Hwa et al., 1991), however the following empirical characteristics are commonly observed in majority of PDS shape profiles: (a) The amplitude of the depolarization peak (*A*_*DP*_) is usually greater than the amplitude of the afterhyperpolarization peak (*A*_AHP_) (Kandel et al., 2000) and (b) The duration of PDS burst τ_*B*_ is less than or equal to the duration of after-hyper-polarization τ_AHP_, τ_*B*_ ≤ τ_AHP_.

The transformation Γ : (τ_*B*_, τ_AHP_) → (Δ_*RP*_, Δ_*PF*_, Δ_FQ_) from individual PDS events into a mean field IIS event is mediated via synchronization of PDS events, which can be quantified by estimating the distribution in the timing τ_*s*_, of the occurrences of PDS in the CA1 Py cells. In order to characterize this transformation, we use a template based method to first construct an artificial PDS template (ρ) parameterized by τ_*B*_ and τ_AHP_ as follows:

(5) $$\rho (t)=\{\begin{array}{ll} -\frac{et}{\tau_{B}}e^{\frac{t}{\tau_{B}}} & \text{ if t}\leq 0\\ -\frac{\beta et}{\tau_{\text{AHP}}}e^{-\frac{t}{\tau_{\text{AHP}}}} & \text{ if t}>0 \end{array}$$

**Figure 3:**
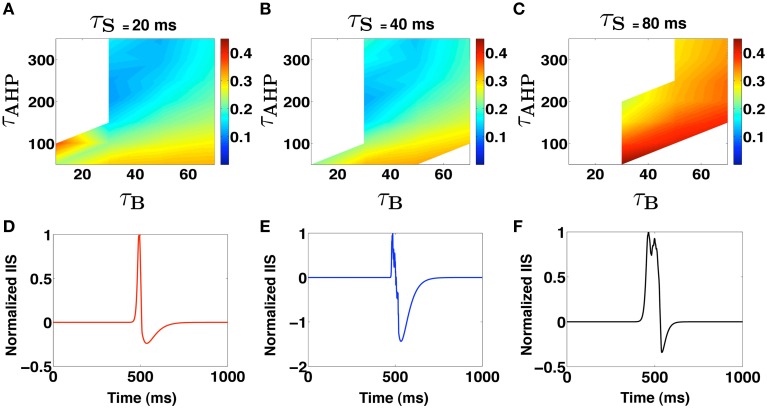
**(A–C)** Shows regions in the PDS template parameter space where IIS occurs. White spaces indicate absence of valid IIS while the colors code for the RMSE of the template generated IIS with the Mean experimental IIS. **(D–F)** Show examples of template derived LFP profiles. **(D)** Shows a valid IIS profile while **(E,F)** show cases when τ_*B*_ << τ_AHP_ and τ_AHP_ << τ_*B*_, respectively.

This empirical function produces a PDS envelope with the PDS depolarization peak normalized to 1 as illustrated in Figure 1D. These artificial PDS constructs are then used to generate a template IIS event as follows:

(6) $${\text{IIS}}_{\text{template}}(t)=\sum_{k}\rho (t-t_{0}+t_{k})$$

where $t_{k}\in \left[\frac{-\tau_{s}}{2},\frac{\tau_{s}}{2}\right]$ represents the time of occurrence of *k*th PDS event relative to a reference time *t*_0_. We note that the parameter τ_*s*_ controls the effective degree of synchronization of the PDS events that are implicated in the generation of the IIS. Using the IIS template in equation 6, we estimate the set of parameter values {τ^*^_B_, τ^*^_AHP_, τ^*^_s_} that produce an IIS template with a “closest-fit” match [root mean squared error (RMSE) <0.1] to the experimentally recorded mean field IIS (Figure 1A). The key results of our template based analysis are summarized in Figure 3. In Figures 3A–C, we plot the RMSE as function of τ_*B*_ and τ_AHP_ for three specific values of τ_*s*_ = {20, 40, 80} ms, respectively. In Figure 3D, we present an example of a valid IIS template event with the closest match (lowest RMSE) to the experimental IIS. In Figures 3E,F, we show examples of invalid IIS template events generated using PDSs with τ_*B*_ << τ_AHP_ and τ_*B*_ >> τ_AHP_, respectively. From Figure 3A we identify PDS parameters {τ_*B*_, τ_AHP_, τ_*s*_} = {50 ms, 350 ms, 20 ms} that produces a template IIS with low RMSE against experimental IIS(≈ 0.1). We use these parameters as initial conditions and minimize the template IIS RMSE using the Nelder-Mead simplex method (Lagarias et al., 1998) to obtain τ^*^_B_ = 37 ms, τ^*^_AHP_ = 300 ms, and τ^*^_s_ = 24 ms.

### 3.2. Estimating the synaptic parameters for the schaffer collateral afferents

**Figure 4:**
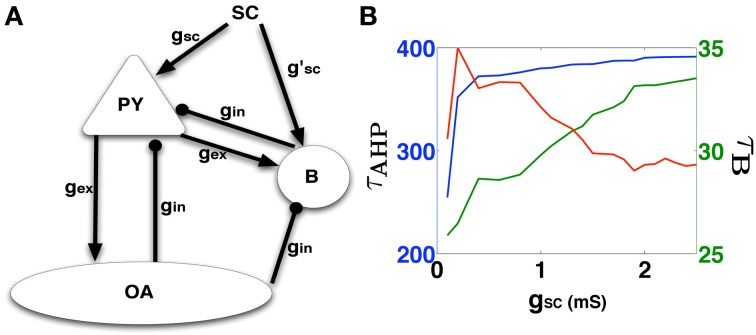
**(A)** The synaptically reduced CA1 network consisting of the basic neuron types with synaptic interconnections (except autapses). The network is used to determine synaptic parameters (*g*_*i*_ and *g*_*sc*_) that produces a PDS matching that predicted by the template studies. **(B)** Shows the variation of burst width (τ_*B*_) in green, AHP width (τ_AHP_) in blue, and the RMSD error (red curve which is scaled by a factor of 30 to match the green Y-axis on the right) of these values from the template-analysis PDS parameters, as a function of *g*_*sc*_.

In this section we present results of our analysis of a synaptically reduced model for the CA1 subfield that is used to estimate the excitatory synaptic strength of SC input onto the epileptic CA1 Py cells. The SC-Py synaptic strength is chosen such that the Py cell elicits PDS bursts with measured temporal features (τ_*B*_,τ_AHP_) matching those obtained from the template PDS analysis. We define the synaptically-reduced model of the CA1 subfield by incorporating just the essential connectivity patterns between a single CA1 Py neuron and the two prominent interneuron types; the basket cell (B) and the orien-alveus cell (OA). The synaptically-reduced network's architecture is illustrated in Figure 4A. The conductance values for all synapses (Py − B = Py − OA = *g*_*ex*_ and SC − B = B − Py = OA − Py = *g*_*in*_) except for SC − Py = *g*_*SC*_, are estimated by matching the PSP magnitudes from each cell type (see Methods: Synaptic models). We then systematically varied *g*_*SC*_, corresponding to the strength of excitatory synaptic input from the SC onto the Py, in order to trigger a template matched PDS response from the CA1 Py cell. The results of these calculations are reported in Figure 4B. We notice that the error in τ_*B*_ and τ_AHP_ values decreases and saturates between 1.5 ≤ *g*_*SC*_ ≤ 2.5 such that the synaptically reduced network elicits a PDS with temporal features {τ^*^_B_, τ^*^_AHP_} ≈{37, 370} ms, which conform with the parameters identified for the template based PDS event. An example of this model generated PDS is shown in Figure 1C. These synaptic parameters are next used in the construction of a biophysically relevant CA1 network model that is capable of generating IISs which matches the features of experimentally recorded IISs.

### 3.3. Eliciting IIS from the CA1 network model

The synaptic parameters obtained from the synaptically-reduced network model of CA1 neurons were incorporated into a biophysically relevant network model of the CA1 subfield comprising of excitatory Py neurons (≈230) and two major interneuronal subtypes, the basket (B) cells (≈30) and the orien-alveus (OA) cells (≈30). All the neurons were modeled as single compartment model neurons following the conductance based Hodgkin-Huxley framework. To address the issue of variability in biological neurons and its effect on system dynamics (Marder and Taylor, 2011), in particular the formation of IIS, we investigated two different single compartment CA1 Py neuron models in our construction of the CA1 network. Interneuronal dynamics are implemented using well-established single-compartmental neuron models, the Wang-Buzsaki model for fast spiking basket cells (Wang and Buzsáki, 1996) and the Wang bursting neuron model for the OA cells (Wang, 2002). Further details on the neuron model types and the distribution of synaptic connectivity in the network are provided in the Methods Section. Using this model we investigate how pathological changes in the CA1 subfield including sprouting of CA1 Py neurons and the variability in the afferent input from the SC affects the CA1 subfield's ability to elicit IISs. Toward this end, we systematically investigated the likelihood for CA1 to elicit an IIS as a function of (a) the degree of synchronization in the volley of afferent input from SC onto the CA1 neurons. (b) the percent of CA1 Py cells that receive direct afferent input from the SC and (c) the degree of CA1 Py neuronal sprouting.

**Figure 5:**
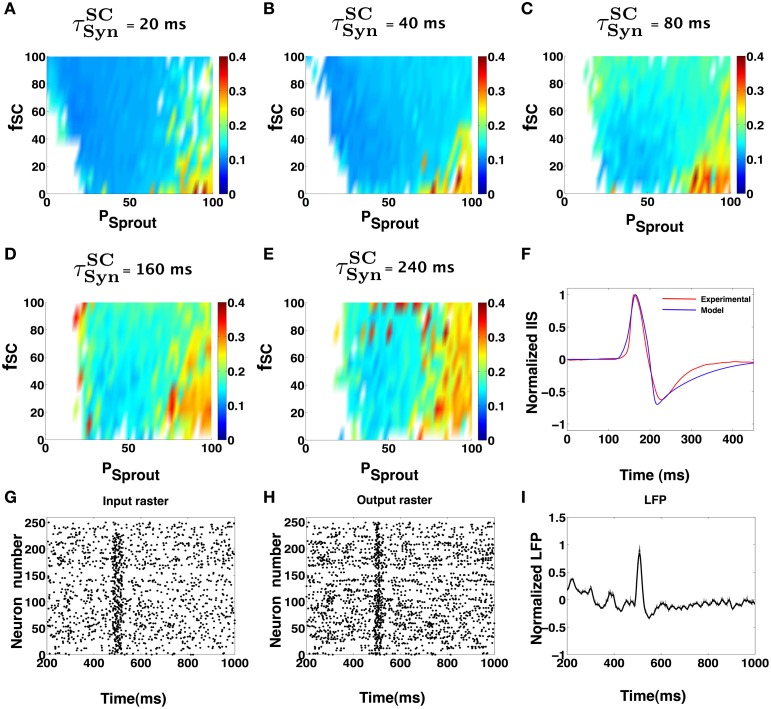
**(A–E)** Show IIS RMSE with the mean experimental IIS, color coded maps in terms of network parameters and input synchronization (jitter). In each of the colormaps for a fixed input window (τ^*SC*^_Syn_), the input percentage from SC to pyramidal cells (*f*_*SC*_), and the degree of sprouting (*P*_sprout_) was varied. **(F)** Shows an example model generated IIS in comparison with the mean experimental IIS. **(G–I)** Shows the input and output rasters and the LFP measured from the activity of the CA1 during low sprouting. The Py cells receive low frequency Poisson input via SC but at 500 ms, there is a quasi-synchronous barrage of input that causes the Py in the CA1 to fire in a synchronous manner. This activity is an example snapshot of the multiple simulations done to create **(A–E)**.

The degree of synchronization in the afferent spike volley was varied as a function of a temporal window τ^*SC*^_Syn_ in which a given fraction *f*_*SC*_ of CA1 neurons receive afferent input from SC with uniform probability. The degree of sprouting was quantified in terms of percent sprouting *P*_sprout_, corresponding to the average number of synapses a given CA1 Py cell receives from other Py cells in the network. For example, *P*_sprout_ = 40 implies any given CA1 Py neuron in the network receives synaptic input from 40 other CA1 Py neurons in the network. In Figures 5A–E, we summarize the key results of our simulation studies to identify the set of parameter values α = {τ^*SC*^_Syn_, *f*_*SC*_, *P*_sprout_} for the CA1 network that can trigger an IIS that satisfies the empirical criteria for an experimentally observed IIS. The color code corresponds to the RMSE between the simulated IIS and the mean profile of the experimentally recorded IIS. The white space in the color plots correspond to the regions in the parameter space α, where the CA1 network failed to trigger an IIS that satisfied the empirical criterion. In Figure 5G, we show a spike raster plot of a typical input received by CA1 Py cells from the SC and in Figure 5H, we show the spike raster of the response of CA1 Py neurons to the SC input. Finally, in Figure 5I, we show the corresponding LFP activity including the presence of an IIS that is generated by the CA1 network model.

As can be seen from Figures 5A–E, the CA1 network with very low sprouting, mimicking the topology of a normal brain, will not elicit an IIS except when a large fraction of CA1 neurons (*f*_*SC*_ ≥ 80%) receives a quasi-synchronous barrage of afferent input (τ^*SC*^_Syn_ ≤ 20 ms) via SC. Increased recurrent connectivity of CA1 (*P*_sprout_ > 40) can evoke IIS in the CA1 network even in the presence of low synchrony SC afferent input (τ^*SC*^_Syn_ > 80 ms) and a low fraction of SC input to CA1 pyramidal cells (*f*_*SC*_ ≈ 30%). This suggests that the ability of CA1 to trigger an IIS increases with increasing recurrent connections in the network and is less dependent on the variability in the afferent input from the SC. In turn, this indicates that IISs may become more frequent with increased axonal recurrent sprouting as a wider range of SC input is now sufficient to elicit an IIS. Considering a previous hypothesis, that the epileptic CA1 pyramidal axonal sprouting increases over time and enhances the CA1's ability for local recruitment during population bursts (Smith and Dudek, 2001), our computational observations suggests that the frequency of IIS events may increase over time during epileptogenesis. Indeed, this phenomenon has been reported in animal models of epilepsy (Buzsáki et al., 1991).

**Figure 6:**
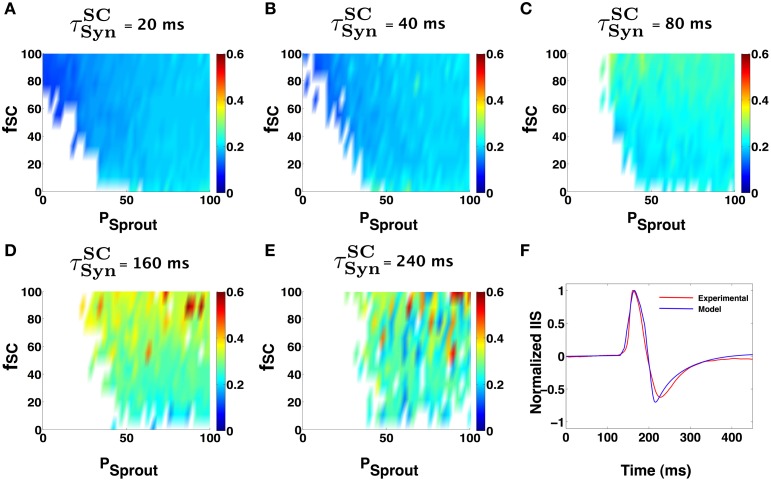
**Similar network parameter IIS trends seen in the case of the Golomb Py model (Figure 5), can be observed using the Nowacki model for the bursting Py cells**. **(A–E)** Show IIS RMSE with the mean experimental IIS, color coded maps in terms of network parameters and input synchronization (jitter). In each of the colormaps for a fixed input window (*t*^*SC*^_Syn_), the input percentage from SC to pyramidal cells (*f*_*SC*_), and the degree of sprouting (Psprout) was varied. **(F)** Shows an example model generated IIS in comparison with the mean experimental IIS.

We next investigated how the results presented above vary with the choice of the CA1 Py model neuron used in the implementation of the CA1 network. We employed a CA1 pyramidal cell model, recently developed and validated by Nowacki et al. (2011). Figures 6A–E show that our general conclusion for the region in the parameter space of α where the CA1 network can trigger an IIS remains unaltered. Minor differences in the degree of Py sprouting and the percent of CA1 neurons receiving direct synaptic input from SC is attributed to the difference in the intrinsic excitability of the model Py neurons. Following from these findings we observe that the ability to trigger an IIS in an excitable CA1 network is primarily dependent on the ability of an individual CA1 neuron to generate a PDS like burst in response to synaptic input from SC and is less dependent on the exact details of the mechanism of the PDS generation itself. Figure 6F compares an IIS generated using the Nowacki model for PY in the CA1, against the mean experimental IIS.

**Figure 7:**
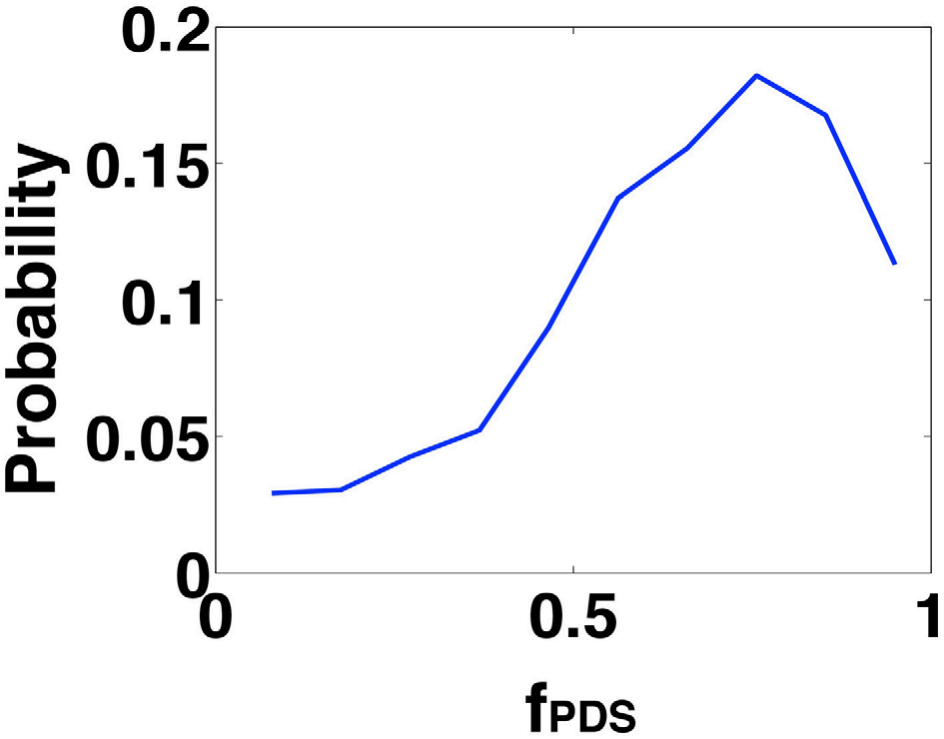
**Shows the probability distribution function of the fraction of the PDS events (*f*_*PDS*_) that fall within a 24 ms window(as predicted by the template studies) during an IIS event**. It can be seen that the probability is much higher for fractions greater than 50%.

Finally, we investigated the degree of PDS synchronization implicated in the generation of an IIS. From the template based analysis, we predicted that an IIS will result when individual PDS events occur within a temporal window of τ^*^_s_ = 24 ms. In order to verify the significance of this prediction, we analyzed the distribution of PDS events generated in the CA1 network in response to afferent drive from the SC. Specifically, for each IIS event triggered in the CA1 network, we look for the fraction of PDS events (*f*_*PDS*_) that fall within a temporal window of ±12 ms around the peak of the triggered IIS event. In Figure 7, we plot the distribution of this fraction. We find that when an IIS occurs, on average ≥50% of the PDS events generated in the network lie within a 24 ms temporal window, in agreement with the PDS empirical estimation results.

### 3.4. Spontaneous generation of IIS from high recurrence and sparse random input

**Figure 8:**
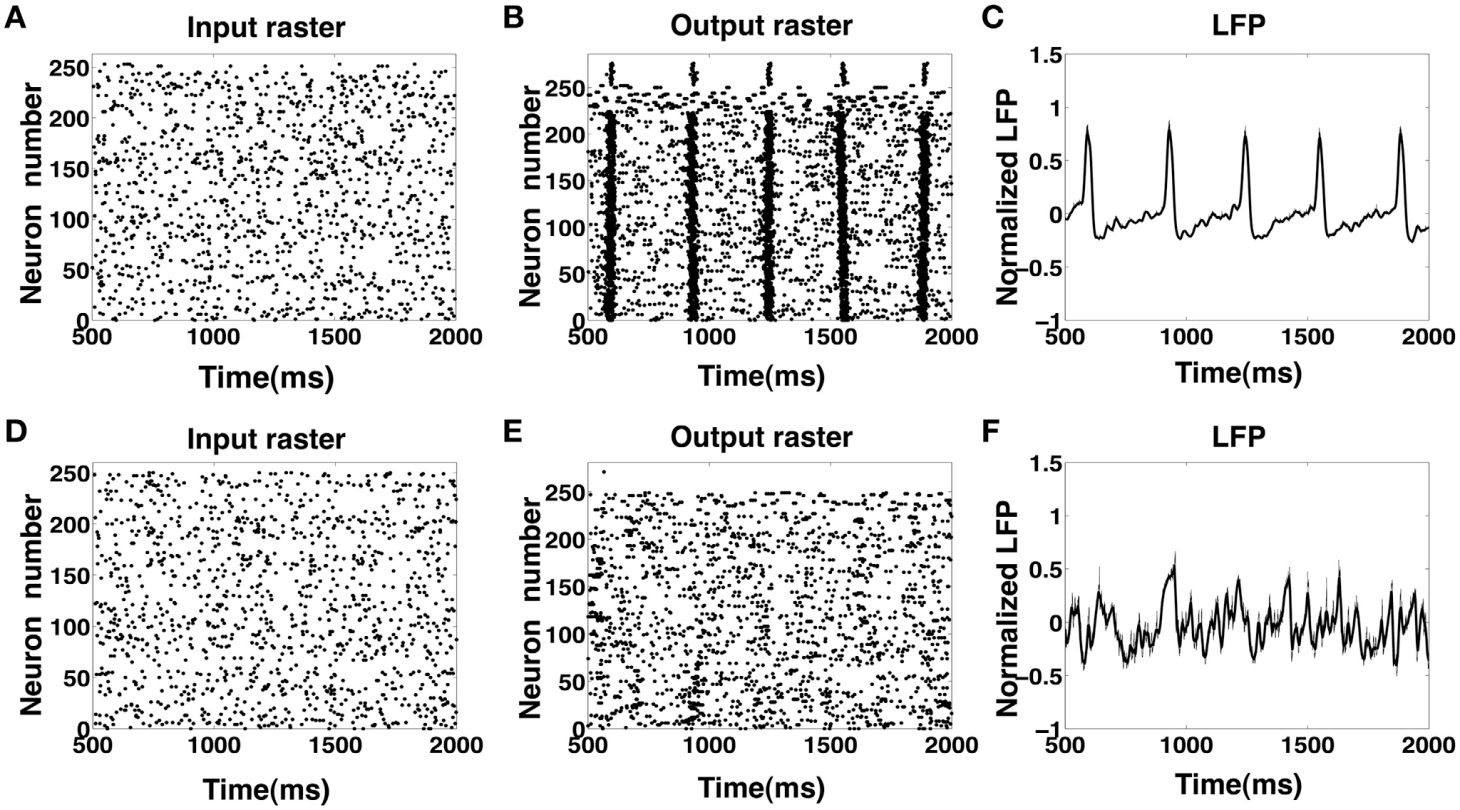
**(A–C)** Input and output rasters and the LFP from CA1 network activity with high sprouting(*P*_sprout_ ≈ 70). **(D–F)** Show input and output rasters and LFP of network activity when sprouting is low (normal case).

**Figure 9:**
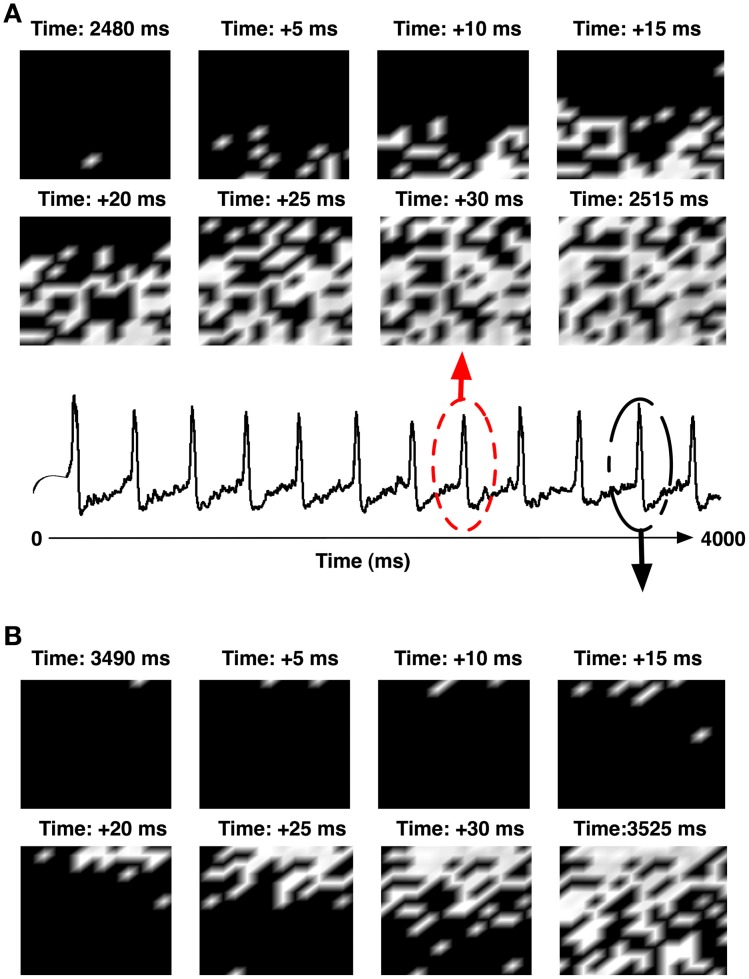
**(A)** Shows the pattern of network synchronization during the IIS event at 2480 ms (circled in red). **(B)** The synchronization of another IIS at 3490 ms (circled in black). The IISs were generated in a network with high recurrent sprouting (*P*_sprout_ ≈ 70).

Motivated by our finding that the CA1 network with a high degree of recurrent connections is able to elicit an IIS even in the absence of a synchronous barrage of afferent input from the SC, we investigated the response of the CA1 network to an asynchronous afferent input from the SC modeled as a sequence of identically independently Poisson distributed spikes occurring at 5 Hz. The results are summarized in Figures 8A–C. We observe that in response to Poisson distributed random SC input, the CA1 network, with sufficiently high degree of sprouting (*P*_sprout_ > 65) responds by emitting a periodic sequence of spontaneously triggered IIS occurring at ≈3 Hz. This type of quasi-periodic synchronization in response to random input have been observed in generic networks of coupled excitatory and inhibitory neuronal populations(van Vreeswijk and Hansel, 2001; Kudela et al., 2003). On further investigation, we noticed that the spread of network activity during a population burst corresponding to these spontaneously triggered IIS events was not fixed. This is illustrated in Figure 9. In the two selected IISs from the same simulation, the spread of synchronized bursting can be seen occurring in opposite directions. This observation suggests that specialized neurons (like hub neurons) may not be necessary for this kind of burst synchronization. We also decreased the percentage of recurrent excitatory sprouting and as suggested from the reported findings in the previous section, all IIS activity in the network was abolished (Figures 8D–F).

**Figure 10:**
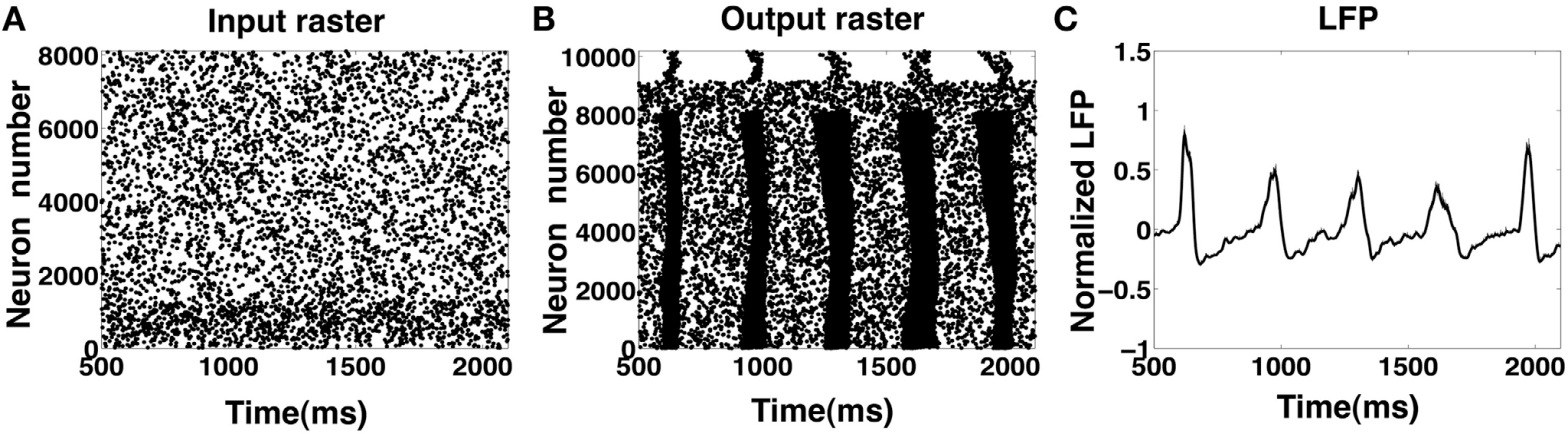
**(A–C)** Input and output rasters and the LFP from the large scale CA1 model (≈10,000 neurons). The scaled up network still retains similar dynamics and IIS waveforms as in the smaller scale.

We also investigated the effect of scaling on the network's ability to exhibit spontaneous periodic IIS activity by increasing the size of the CA1 network 30 fold (by scaling the network in X and Z dimensions shown in Figure 2B) while maintaining the network connectivity, the level of neuronal excitability and the synaptic strengths. The scaled network consisted of 8000 pyramidal cells, 1000 basket, and 1000 oriens/alveus cells. The sprouting was maintained at approximately *P*_sprout_ = 65. The network was stimulated with 5 Hz Poisson distributed random input via SC. The scaled network's rasters and LFP responses are shown in Figures 10A–C. We observed that the scaled model exhibited identical behavior to that of the original model in that IIS were generated at a similar rate of ≈3 Hz in the scaled network as well.

### 3.5. IIS formation as an induced or emergent synchronization phenomenon

**Figure 11:**
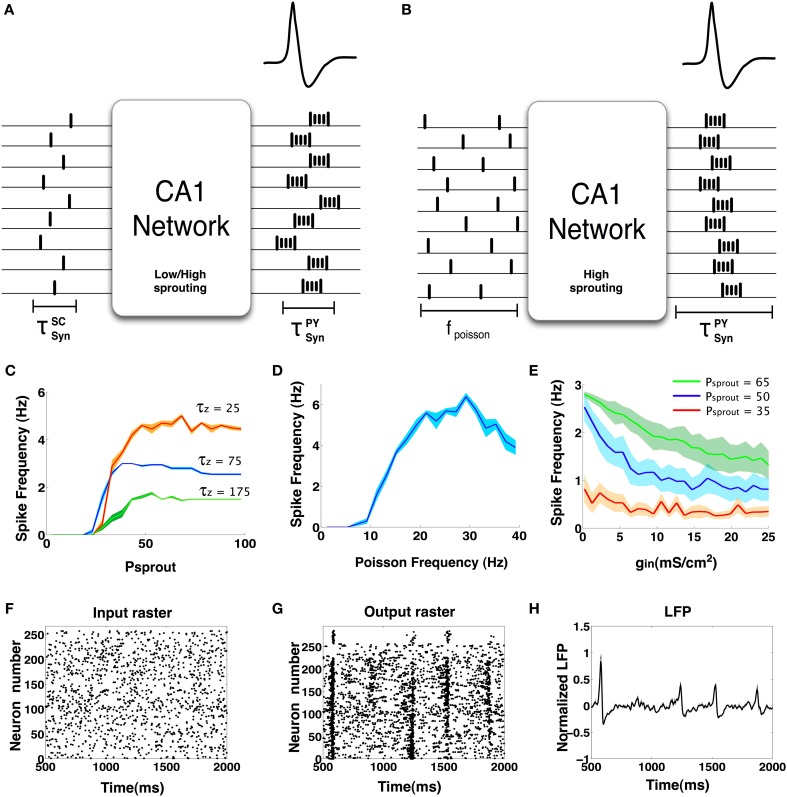
**(A)** This figure schematically represents the concept of induced synchrony: When an already synchronous barrage of input arrives at the CA1 network, the output also tends to be synchronous in nature. This has been observed in networks with both low and high degree of sprouting. **(B)** Another case when IIS is possible but without synchronous input. In this case we observe that the CA1 network must have a high degree of sprouting and receive sparse random input continuously. **(C)** IIS frequency vs. sprouting trends. We see that all IIS frequencies tend to saturate by 100% sprouting. Lower values of the M-current decay constant (τ_*Z*_) significantly changes the maximum rate of spontaneous IIS exhibited in the network. The mean trend is shown in dark colors while the lighter bands indicate the standard error. **(D)** The nature of IIS rate vs. Poisson input frequency is non- monotonic. Networks that are less excitable (*g*_*sc*_ = 1.0 mS/cm^2^, *I*_*DC*_ = 0.1 μA/cm^2^, *P*_sprout_ = 30) show an increase in IIS rate up to a certain point (30 Hz) beyond which the nature of the poisson input goes from synchrony-conducive excitability to synchrony-disruptive excitability. The mean trend is shown in dark blue and the standard error is depicted in the light blue band. **(E)** Increasing GABA synaptic strengths can interfere with synchronization and hence decrease the rate of spontaneous IISs. The mean trend is shown in dark blue and the standard error is depicted in the light blue band. **(F–H)** Input and output rasters and the LFP of a network with high GABA synaptic strength (*g*_*in*_ = 25 ms/cm^2^).

From results presented in the previous Sections, we see that the CA1 network can exhibit IIS via (a) induced synchronization of the PDS events elicited from CA1 Py neurons in response to synchronous barrage of afferent input from the SC and (b) emergent synchronization of PDS events elicited from CA1 Py neurons with high degree of recurrent connections in response to asynchronous Poisson distributed SC input. In Figures 11A,B, we show a schematic diagram depicting the scenarios that can trigger the emergence of IIS in the CA1 network. The mechanism of induced synchronization leading the emergence of IIS is relatively straightforward and does not require recurrent connections between CA1 Py cells as a necessary condition. In the absence of any recurrent CA1 Py neuronal connections, if a large fraction of CA1 Py cells receive sufficiently synchronized SC input; the CA1 Py cells will respond with a high probability of eliciting a PDS more or less simultaneously, resulting in the observation of an IIS in the mean field CA1 network activity.

On the other hand, the spontaneously occurring IIS in the CA1 network in response to asynchronous Poisson distributed SC input requires recurrent CA1 Py connections as a necessary condition. We refer to this phenomenon as emergent synchrony of the PDS events because the synchrony emerges from within the network as opposed to primarily from the input. In addition to recurrent connections between the CA1 Py neurons, a number of additional network parameters that regulate the network excitability are implicated in the emergence of spontaneous IIS within the CA1 network. In particular, the strength of inhibitory synaptic connections and the frequency of Poisson distributed SC input play major roles in the emergence of spontaneous IIS.

We begin by analyzing the role of sprouting in the emergence of spontaneous IIS. We systematically varied the degree sprouting (recurrent connections between the CA1 Py neurons) from *P*_sprout_ = 0 to *P*_sprout_ = 100, while keeping all other CA1 network parameters in the default state (see Methods). In Figure 11C, we plot the result of these calculations (shown in blue trace) by measuring the rate of spontaneous IIS as function of the degree of sprouting in the CA1 network. We observe that spontaneous IIS emerge in the network when *P*_sprout_> 25, with the rate of spontaneous IIS saturating to a maximum value of ≈3 Hz for *P*_sprout_ = 100 sprouting in the network. Further investigations led us to the observation that the slow activation time constant τ_*Z*_ of the potassium M-current of Py cells is primarily implicated in determining the maximum rate of spontaneous IIS activity in the CA1 network. This is illustrated in Figure 11C, where we plot the rate of spontaneous IIS in the CA1 network as a function of *P*_sprout_ for different values of τ_*Z*_. We observe that the maximum rate of spontaneous IISs monotonically decreases with increasing value of τ_*Z*_. This is because for smaller value of τ_*Z*_, the M-current activates faster resulting in truncating the number of spikes per burst of PDS activity, while faster deactivation of M-currents result in an increased rate of PDS bursting activity, which in turn triggers the emergence of spontaneous IIS at an increased rate.

**Figure 12:**
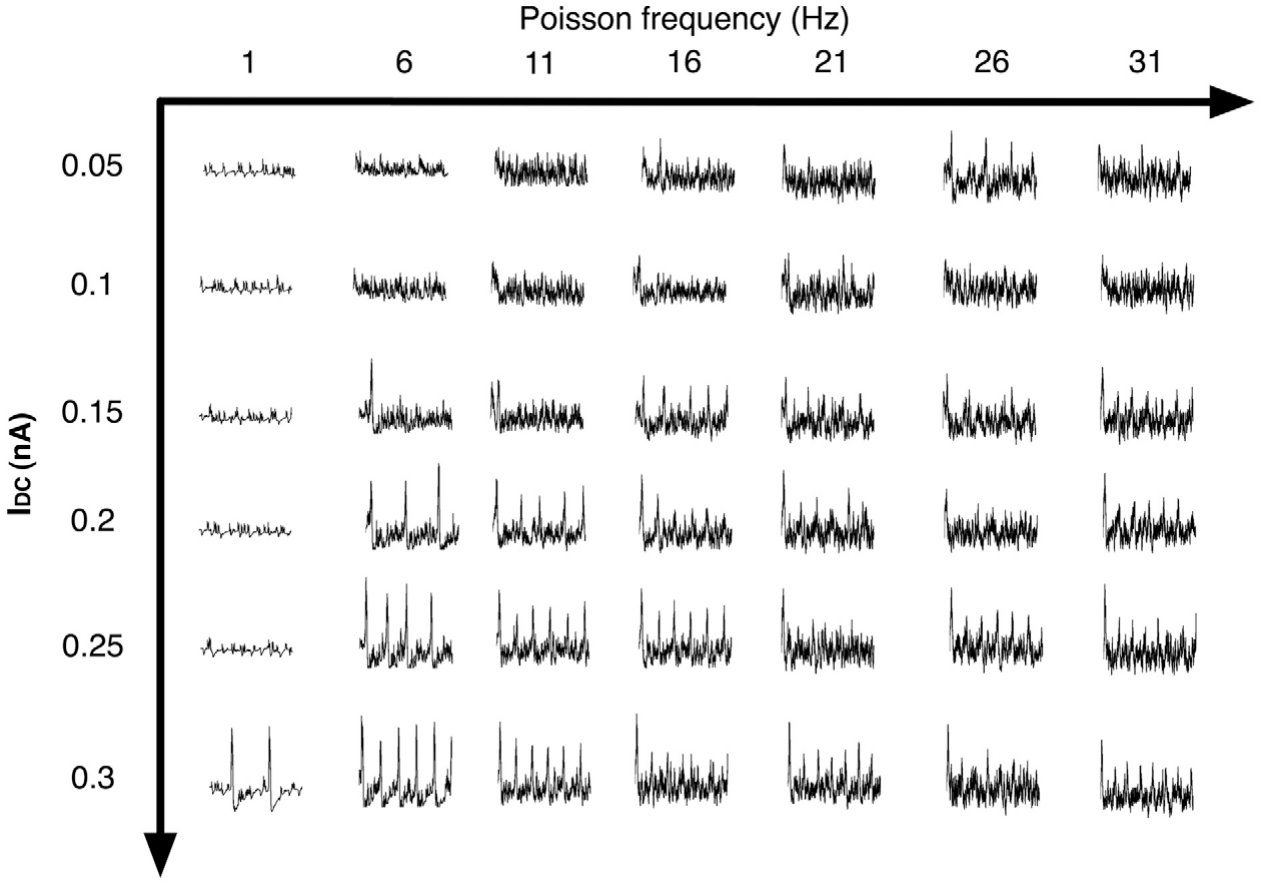
**The figure shows the LFP of the CA1 network for different SC input rates (Poisson frequency varied) and different levels of neuronal excitability (*I*_*DC*_)**. In general as the rate of SC input increases, the network goes from producing IIS-like spikes to higher frequency oscillatory activity.

We next analyzed the dependence of the rate of spontaneous IIS on the frequency of Poisson distributed random SC input. For a default network configuration with *P*_sprout_ = 70, we systematically increased the frequency of Poisson input from 5 Hz to 30 Hz. The results of this analysis are presented in Figure 11D. We see that, with default network parameters, the network initially responds by generating spontaneous IIS at ≈3 Hz, but as the frequency of Poisson input increases, the network exhibits high frequency-low amplitude non-IIS like spiking activity. By estimating the degree of synchronization amongst the CA1 Py neurons using a well-established synchronization measure Hansel and Sompolinsky (1992); Kudela et al. (2003), we find that the network receiving Poisson distributed random SC input at 30 Hz exhibits 70% less synchronization than is the case when the network exhibits spontaneous IIS in response to Poisson distributed random SC input at 5 Hz. This suggested that increasing the rate of random spikes to the CA1 via SC disrupts network synchronization making the network less susceptible to exhibit spontaneous IIS. It should be noted that this finding was observed in a network that was already in an excitable state, i.e. high degree of recurrent connections and Py cells are highly input sensitive (*I*_*DC*_ = 0.3 μA/cm^2^). We next investigated if this finding varied with a network of lower intrinsic network excitability. We reduced the degree of sprouting to 30 and at the same time reduced τ_*Z*_ to 25 ms, such that the network does not exhibit spontaneous IIS at the saturating rate of 6 Hz observable for network with *P*_sprout_ = 100. We also reduced the Py cells intrinsic excitability (*I*_*DC*_ = 0.1 μA/cm^2^, *g*_*sc*_ = 1.0 mS/cm^2^). In this case, as we increased the frequency of Poisson distributed random SC input, the rate of spontaneous IIS activity is increased reaching a peak of 6 Hz for Poisson input frequency of 30 Hz. Thus, depending on the level of network excitability the rate of random SC input can be either conducive or disruptive to emergent synchronization in the network. In Figure 12 we show examples of LFPs generated by the CA1 network model for different values of SC input rates and excitability levels of Py cells (*I*_*DC*_). We notice that in general as the rate of SC input increases the network goes from producing IIS-like spikes to higher frequency oscillations.

Finally, we analyzed how the rate of spontaneous IIS depends on the strength of inhibitory synaptic connections in the network. For different values of *P*_sprout_ = {35, 50, 65}, we gradually increased the strength of inhibitory synaptic couplings in the network. From Figure 11E we see that the frequency of spontaneous IIS decreases with increase in network inhibition and eventually saturates. Furthermore, we also observe that the rate at which the IIS frequency saturates depends upon the degree of sprouting of pyramidal cells in the CA1 network. We see that for *P*_sprout_ = 65, the frequency of spontaneous IIS takes longer to saturate than in the case of *P*_sprout_ = 35 where the frequency of IISs saturates to a non-zero minimum (<1 Hz) much faster. Finally, we make the observation that when *P*_sprout_ is sufficiently high to induce spontaneous IISs, even high values of GABAergic conductances are unable to completely eliminate IISs. This possibly suggests that when a CA1 network reaches a sufficiently hyperexcitable state through recurrent pyramidal cell sprouting, even enhanced interneuronal activity may be insufficient in completely suppressing epileptiform activity (Franck and Schwartzkroin, 1985; Franck et al., 1988; Bausch, 2005). In Figures 11F–H, we show an example of raster and LFP plots when GABA strength (*g*_*in*_) values were set at 25 mS/cm^2^. The Figures clearly show that spontaneous IISs can still occur with high network inhibition but many of the synchronization attempts of the Py cells are thwarted by interneuronal interferences. This results in much weaker islands of synchronization (Figure 11G at 1500 and 2000 ms) or no synchronization at all (at 900 ms).

## 4. Discussion

Our primary goal was to first develop a biologically relevant platform for modeling IISs in the CA1, and then to analyze the network conditions necessary for IISs. In order to build the CA1 network model such that it possesses the capability to exhibit IISs, we had to consider various cellular and network level parameters possibly implicated in the role of IIS genesis. In order to avoid the impracticality of a computationally expensive parametric exploration of a high dimensional system for IIS generation, we chose to dissociate the cellular level parameters from network level parameters and analyze them separately in a hierarchic fashion.

We introduced a template based approach for estimating the empirical features of the cellular correlate of IIS, i.e., the PDS burst width (τ_*B*_) and the PDS after-hyperpolarization duration (τ_AHP_), from experimentally recorded IIS. These features were used to estimate the synaptic parameters of a reduced CA1 network, which in turn allowed us to develop a biologically relevant model of the CA1 network capable of generating an IIS event with empirical characteristics matching those obtained from experimental recordings from the CA1 of an *in vivo* animal model of epilepsy. We believe that the proposed approach, leveraging experimental data to estimate network parameters, may be used for the analysis and development of models for other LFP features besides IIS. We however note that the template based approach proposed here provides a ballpark estimate for some of the critical network parameters. For example, PDSs that occur during an IIS would most likely have a distribution of burst width and AHP durations with the mean values presumably close to our estimated values. To our knowledge not many other methods are currently available for estimating computational network parameters solely from experimental recordings of LFP data such as IIS. This step is important for the biological validation of computational models that attempt to capture experimental features of interest.

We next developed a biophysically relevant model of the CA1 network in order to identify the network conditions under which the CA1 network can elicit an IIS. We found that the CA1 can trigger an IIS event under a variety of conditions. For network configurations characterized by a low degree of sprouting, IISs can be evoked by a synchronous barrage of afferent input from the SC with high percentage of SC to Py connectivity. The simulations also indicated that many pyramidal cells (>80%) are recruited via the SC when an IIS is triggered. For higher degrees of sprouting, we found that the formation of IIS is less dependent on the degree of input synchronization and the percentage of SC to Py connections. Indeed, even in the presence of low input synchronization (τ^*SC*^_Syn_ = 240 ms) and low *f*_*SC*_, we noticed that IISs could still form for a sufficiently large degree of CA1 sprouting (*P*_sprout_ ≥ 40). These findings suggest that sprouting may play a significant role in synchronization of PDSs, resulting in the manifestation of an IIS. We also observed that in the presence of sufficiently high degree of Py cell sprouting, sequence of asynchronous afferent input onto the CA1 Py cells from the SC can trigger spontaneously generated IIS events that occur in periodic fashion. These results indicate that the CA1 network is less influenced by the nature of SC input as the degree of Py sprouting increases. If we consider the hypothesis suggested by Staley et al. (2005) that sprouting is a phenomenon that progresses over a period of weeks in the TLE brain, then in conjunction with our results this indicates that IISs will gradually increase in their frequency over a timespan. This phenomenon was in fact observed experimentally by Buzsáki et al. (1991).

The CA1 network is capable of producing IISs primarily through two mechanisms of synchronization, (a) input-induced synchronization and (b) emergent synchronization. We found that while induced synchronization is a straightforward manifestation of SC input on Py output synchronization, emergent synchronization was a more complex phenomenon. IIS formation by emergent synchronization seems to depend on the CA1 network's intrinsic excitability. The level of the CA1 network excitability depends on many parameters. We observe that when the CA1 network has achieved a sufficient degree of Py cell recurrent sprouting, even enhanced GABAergic strengths cannot completely suppress epileptiform activity. This observation is in agreement with prior experimental studies (Franck and Schwartzkroin, 1985; Franck et al., 1988) where enhanced GABAergic activity alone was insufficient in controlling epileptic hyperexcitability. We also see that if the network is already in an excitable state (primarily high recurrent Py sprouting and sufficiently excitatory synaptic strengths) and is capable of exhibiting IISs, increasing the SC drive significantly (30 Hz) to the CA1 may actually disrupt IISs and instead result in high amplitude oscillatory activity. Hence there is a domain of network excitability in terms of SC input and network parameters for which IIS formation is possible.

We note that NMDA synapses were not incorporated in our implementation of the CA1 model primarily because it has been demonstrated that NMDA synapses are not critical for burst initiation in the hippocampus when the brain is already in an epileptic state (Stoop and Pralong, 2000; Stoop et al., 2003). It may, however, be worth investigating the effects of NMDA on long-term changes in the CA1, such as sprouting and synaptic plasticity between recurrent pyramidal cells.

**Figure 13:**
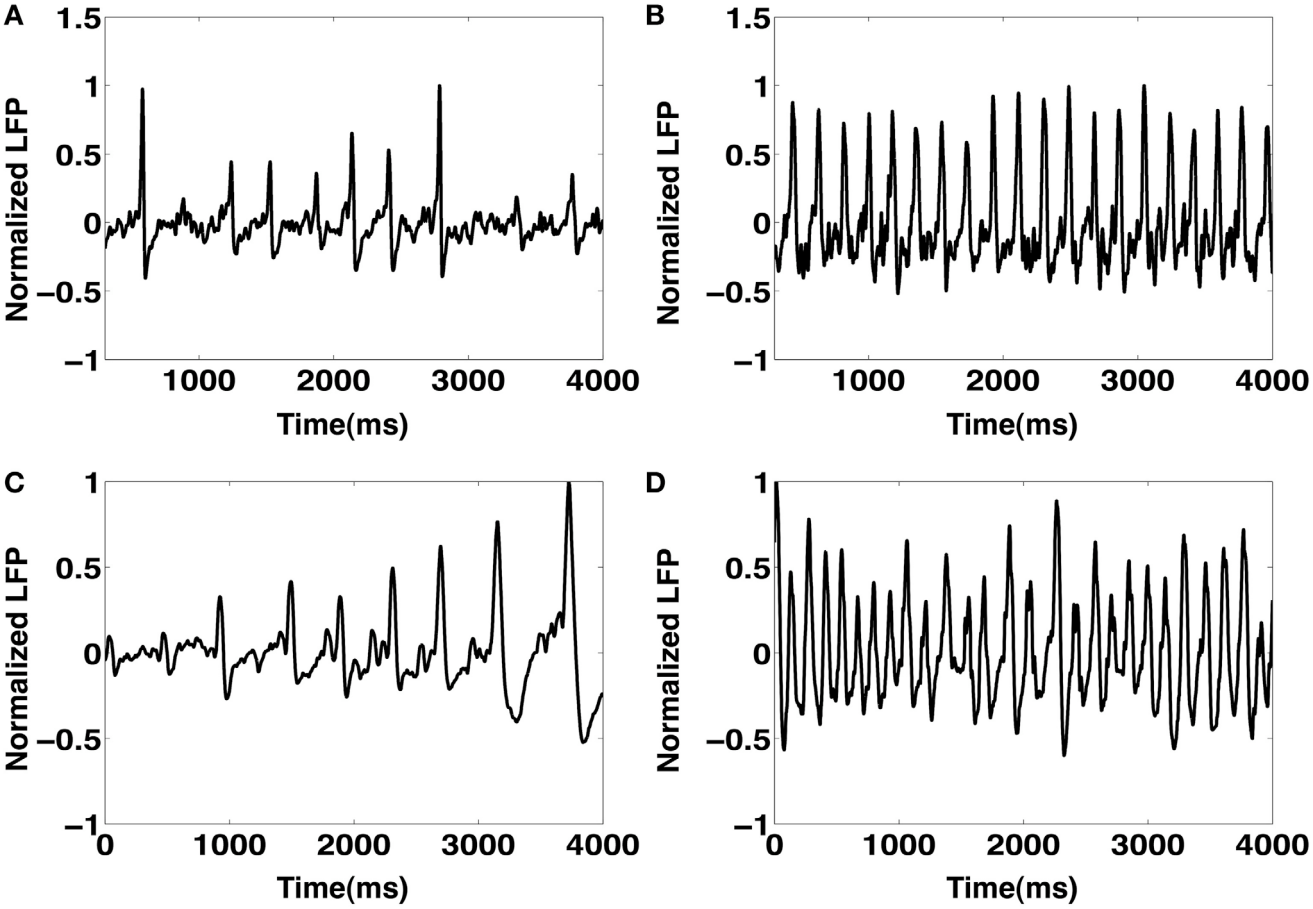
**(A)** A sequence of spontaneous IIS-like events produced from the CA1 network model. **(B)** An example of the CA1 model generated tonic ictal-like activity in the theta range. This is primarily produced by increasing the frequency of SC input drive to the CA1 (@ 30 Hz). **(C)** Sequence of IIS events observed in an EEG recording of a rat induced with TLE. **(D)** EEG recordings of the tonic phase of an ictal event.

While IISs have been the focus of this work, the CA1 network model may be capable of exhibiting other significant LFP patterns. For instance, we observe that in the event of significantly increased random input drive from the SC (@ 30 Hz), the CA1 network with a high degree of sprouting produces non-IIS oscillatory activity in the theta range (4–8 Hz). This LFP activity is extremely similar to the tonic phase of an ictal event (Quiroga et al., 1997). We illustrate comparisons between our model's LFPs and experimentally recorded data for the IISs and the tonic phase of an ictal event in Figure 13. Figures 13A,C show IISs generated from the model CA1 network and from experimentally recorded EEG, respectively. Figure 13D shows the EEG recording of the tonic phase of an ictal event. In comparison, the model is able to generate a similar LFP waveform as shown in Figure 13B when the rate of Poisson SC input is increased to 30 Hz. We anticipate that our modeling paradigm may serve as a framework for future investigators interested in incorporating further details in the CA1 model in order to better understand the mechanisms of epileptogenesis.

### Conflict of interest statement

The authors declare that the research was conducted in the absence of any commercial or financial relationships that could be construed as a potential conflict of interest.

## Figures and Tables

**Figure A1:**
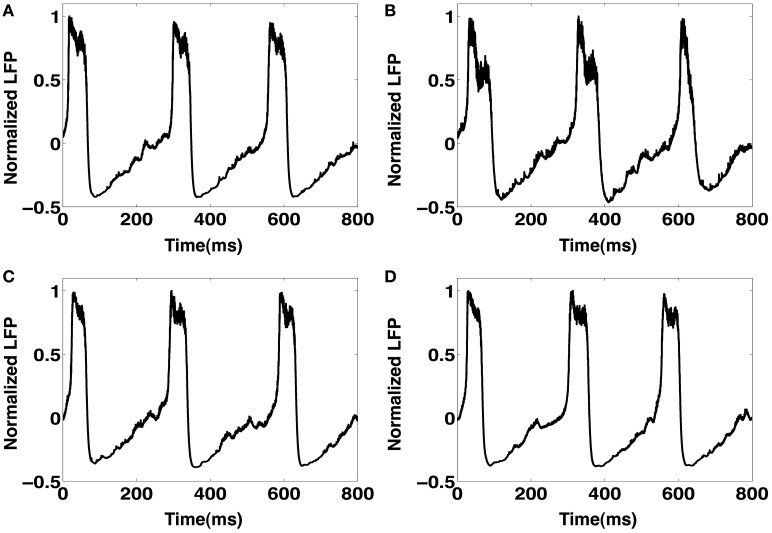
**(A)** An example LFP trace of spontaneous IIS using the Euler integration method for a time step of 0.01 ms. **(B)** An LFP trace using the Euler integration method for a time step of 0.05 ms. **(C)** Spontaneous IIS LFP obtained via simulations using the fourth order Runge-Kutta integration method with a time step of 0.01 ms. **(D)** A similar LFP trace obtained using the fourth order runge-kutta integration method for a timestep of 0.05 ms. All simulations were run with default model parameter values and *P*_sprout_ = 65.

## Appendix

### Neuron model details

In this section we provide details on the channel currents and conduction parameters for the three neuron models described in the methods section. The channel currents for the Golomb model (Golomb et al., 2006) are: *I*_*g*_ = {*I*_*Na*_, *I*_*KDR*_, *I*_*NaP*_, *I*_*L*_, *I*_*A*_, *I*_*M*_} and *I*_*KCa*_ = 0 where,

(7) $$\begin{array}{l} I_{Na}=g_{Na}m_{\infty }^{3}h(V-E_{Na})\\ I_{L}=g_{L}(V-E_{L})\\ I_{KDR}=g_{KDR}n^{4}(V-E_{K})\\ I_{NaP}=g_{NaP}p_{\infty }^{3}h(V-E_{Na})\\ I_{A}=g_{A}a_{\infty }^{3}b(V-E_{K})\\ I_{M}=g_{M}z(V-E_{K}) \end{array}$$

The gating variables are {*n*, *h*, *b*, *z*, *m*_∞_, *p*_∞_, *a*_∞_}, where

(8) $$\begin{array}{l} \dot{h}=\phi (\Gamma (V,\theta_{h},\sigma_{h})-h)/(1+7.5*\Gamma (V,\tau_{th},-6.0))\\ \dot{b}=(\Gamma (V,\theta_{b},\sigma_{b})-b)/\tau_{b}\\ \dot{n}=\phi (\Gamma (V,\theta_{n},\sigma_{n})-n)/(1+7.5*\Gamma (V,\tau_{tn},-15.0))\\ \dot{z}=(\Gamma (V,\theta_{z},\sigma_{z})-z)/\tau_{z}\\ m_{\infty }=\Gamma (V,\theta_{m},\sigma_{m})\\ p_{\infty }=\Gamma (V,\theta_{p},\sigma_{p})\\ a_{\infty }=\Gamma (V,\theta_{a},\sigma_{a}) \end{array}$$

where, ϕ = 1 and $\Gamma (V,\theta ,\sigma )=\frac{1}{1+\exp(\frac{-(V-\theta )}{\sigma })}$ and the parameters are: θ_*m*_ = −30 mV; σ_*m*_ = 9.5 mV; θ_*h*_ = −45 mV; σ_*h*_ = −7 mV; θ_*n*_ = −35 mV; σ_*n*_ = 10 mV; θ_*a*_ = −50 mV; σ_*a*_ = 20 mV; θ_*b*_ = −80 mV; σ_*b*_ = −6 mV; θ_*z*_ = −39 mV; σ_*z*_ = 5 mV; τ_*b*_ = 15 ms; τ_*z*_ = 75 ms; τ_*th*_ = −40.5 ms; τ_*tn*_ = −27 ms.The channels reversal potentials and conductances are as follows: *E*_*Na*_ = 55 mV; *E*_*K*_ = −90 mV; *E*_*L*_ = −70 mV; *g*_*Na*_ = 35 mS/cm^2^; *g*_*NaP*_ = 0 mS/cm^2^; *g*_*KDR*_ = 6 mS/cm^2^; *g*_*L*_ = 0.05 mS/cm^2^; *g*_*A*_ = 1.4 mS/cm^2^; *g*_*M*_ = 1 mS/cm^2^. We set *I*_*DC*_ = 0.3 μA/cm^2^.

For the Wang-Buzsaki model (Wang and Buzsáki, 1996) the channel currents are: *I*_*g*_ = {*I*_*Na*_, *I*_*K*_, *I*_*L*_} and *I*_*KCa*_ = 0, where

(9) $$\begin{array}{l} I_{Na}=g_{Na}m_{\infty }^{3}h(V-E_{Na})\\ I_{L}=g_{L}(V-E_{L})\\ I_{K}=g_{K}n^{4}(V-E_{K}) \end{array}$$

The gating variables are {*n*, *h*, *m*_∞_},where

(10) $$\begin{array}{l} \dot{h}=\phi (\alpha_{h}(1-h)-\beta_{h}h)\\ \dot{n}=\phi (\alpha_{n}(1-n)-\beta_{n}n)\\ m_{\infty }=\frac{\alpha_{m}}{\alpha_{m}+\beta_{m}} \end{array}$$

The gate variables rate constants are given as:

(11) $$\begin{array}{l} \alpha_{m}(V)=\frac{-0.1(V+35)}{\exp(-0.1(V+35))-1}\\ \beta_{m}(V)=4\exp(-(V+60)/18)\\ \alpha_{h}(V)=0.07(\exp(-(V+58))/20)\\ \beta_{h}(V)=1/\exp(-0.1(V+28)+1)\\ \alpha_{n}(V)=\frac{-0.01(V+34)}{\exp(-0.1(V+34))-1}\\ \beta_{n}(V)=0.125\exp(-(V+44)/80) \end{array}$$

The model parameters are: *E*_*Na*_ = 55 mV; *E*_*K*_ = −90 mV; *E*_*L*_ = −65 mV; *g*_*Na*_ = 35 mS/cm^2^; *g*_*K*_ = 9 mS/cm^2^; *g*_*L*_ = 0.1 mS/cm^2^; *c* = 1 μF/cm^2^; ϕ = 5. *I*_*DC*_ was set to 0 mV.

The Wang model's (Wang, 2002) channel currents are: *I*_*g*_ = {*I*_*Na*_, *I*_*K*_, *I*_*h*_, *I*_*Ca*_, *I*_*L*_} and *I*_*KCa*_ = *g*_*KCa*_[*Ca*^2+^]/([*Ca*^2+^] + *K*_*D*_)(*V* − *E*_*K*_), where

(12) $$\begin{array}{l} I_{Na}=g_{Na}m_{\infty }^{3}h(V-E_{Na})\\ I_{K}=g_{K}n^{4}(V-E_{K})\\ I_{h}=g_{h}H(V-E_{h})\\ I_{Ca}=g_{Ca}m_{\infty }^{2}(V-E_{Ca})\\ I_{L}=g_{L}(V-E_{L}) \end{array}$$

The gating variables {*n*, *h*, *m*_∞_} follow the same dynamics as described in equations 10 and11. The additional gating variables are $\left\{\dot{H},\dot{\left[Ca^{2+}\right]}\right\}$ which have the following dynamics:

(13) $$\begin{array}{l} \dot{H}=\frac{H_{\infty }-H}{\tau_{H}}\\ H_{\infty }(V)=1/(1+\exp((V+80)/10))\\ \tau_{H}(V)=\frac{20}{\left(\exp((V+70)/20)+\exp(-(V+70)/20\right)}+5\\ \dot{Ca^{2+}}=-\alpha I_{Ca}-[Ca^{2+}]/\tau_{Ca} \end{array}$$

The parameters for {*I*_*Na*_, *I*_*K*_, *I*_*L*_} are the same as those described in the Wang-Buzsaki model. The additional parameters are: *g*_*KCa*_ = 10 mS/cm^2^; *K*_*D*_ = 30 μM; *E*_*Ca*_ = 120 mV; *g*_*Ca*_ = 1 mS/cm^2^; *E*_*h*_ = −40 mV; τ_*Ca*_ = 80 ms; α = 0.002.

### Numerical integration details

In this section we show that the network behavior remains invariant for different numerical integration methods. In paricular we compare the effects of different time steps (0.01 and 0.05 ms) on the CA1 network's model simulation. We also compare the Euler integration method against the fourth order Runge-Kutta method. The results are illustrated in Figure A1. For a fixed value of *P*_sprout_ = 65 and keeping other model parameters at their default values, we ran simulations to compare the numerical integration time steps and methods. In the case of the Euler integration method, we see that a time step of 0.01 ms (Figure A1A) and a time step 0.05 ms (Figure A1B) both produce very similar spontaneous IIS LFP traces. Even in the case of the fourth order Runge-Kutta integration method, we continue to see no noticable deviation between the LFP traces for simulation time steps of 0.01 ms (Figure A1C) and 0.05 ms (Figure A1D). Furthermore, we also do not notice any significant differences between the LFP traces obtained from the Euler and fourth order Runge-Kutta integration methods. These observations suggest that the choice of time step (0.01 ms) for our simulations is valid.
